# Separation of *Mycobacterium abscessus* into subspecies or genotype level by direct application of peptide nucleic acid multi-probe- real-time PCR method into sputa samples

**DOI:** 10.1186/s12879-015-1076-8

**Published:** 2015-08-11

**Authors:** Kijeong Kim, Seok-Hyun Hong, Byoung-Jun Kim, Bo-Ram Kim, So-Young Lee, Ga-Na Kim, Tae Sun Shim, Yoon-Hoh Kook, Bum-Joon Kim

**Affiliations:** Department of Biomedical Sciences, Microbiology and Immunology, Cancer Research Institute, and Liver Research Institute, Seoul National University College of Medicine, 28 Yongon-dong, Chongno-gu, Seoul, 110-799 Republic of Korea; Department of Microbiology, Chung-Ang University College of Medicine, Seoul, 156-756 Republic of Korea; Division of Pulmonary and Critical Care Medicine, Department of Internal Medicine, Asan Medical Center, University of Ulsan College of Medicine, Seoul, Republic of Korea

**Keywords:** *Mycobacterium abscessus*, *Mycobacterium massiliense*, Peptide nucleic acid (PNA), Real time PCR, *hsp65*, Genotype

## Abstract

**Background:**

Recently, we introduced a novel peptide nucleic acid (PNA) multi-probe real time PCR method targeting the *hsp65* gene (*hsp65* PNA RT-PCR) to distinguish *Mycobacterium abscessus* groups.

**Methods:**

Here, we evaluated the usefulness of the *hsp65* PNA RT-PCR for the direct identification of the *M. abscessus* group at the subspecies and genotype levels from sputa samples. The method was applied to total sputa DNA from 60 different patients who were identified as having mycobacterial infections via *rpoB* PCR restriction analysis of the same cultures.

**Results:**

The *hsp65* PNA RT-PCR method had higher sensitivity than the multi-probe real-time PCR assay targeting *hsp65* (HMPRT-PCR) for the detection of *M. abscessus* from sputum [96.7 % (29/30 samples) vs. 70 % (21/30 samples); 100 % specificity].

**Conclusions:**

These results suggest that the PNA-based method is feasible for the detection of *M. abscessus* members not only from cultures but also directly from sputa.

**Electronic supplementary material:**

The online version of this article (doi:10.1186/s12879-015-1076-8) contains supplementary material, which is available to authorized users.

## Background

The *Mycobacterium abscessus* complex represents a group of rapidly growing mycobacteria (RGM) that account for approximately 65–80 % of RGM pulmonary infections [1]. In Korea, the incidence of *M. abscessus* lung diseases has been increasing; these infections account for 70–80 % of RGM-induced lung diseases [2–5].

The taxonomic status of the *M. abscessus* group remains undetermined*.* Recent advances in taxonomic approaches revealed that the *M. abscessus* group could be further divided into three closely related taxa [i.e., *M. abscessus* subsp. *abscessus* (hereafter referred to as *M. abscessus*)*, M. abscessus* subsp. *massiliense* (hereafter referred to as *M. massiliense*) and *M. abscessus* subsp. *bolletii* (hereafter referred to as *M. bolletii*)] [6–8]. Recently, it was reported that *M. massiliense* could be further subdivided into two genotypes (hereafter referred to as Type I and Type II) based on *hsp65* sequence analysis. All of the strains belonging to Type II are distinct in Korean patients due to a rough colony morphotype without any exceptions [9]. A recent complete genome study revealed that the rough colony phenotype of the Type II strains may be due to a large deletion event in the glycopeptidolipid (GPL)-related genes [10, 11]. Furthermore, a recent report based on pulsed-field gel electrophoresis (PFGE) and multi-locus sequence typing (MLST) also revealed taxonomic separation between *M. massiliense* Type II-1 and Type II-2 [12].

*M. massiliense* has been increasingly recognized as an emerging pathogen that causes postsurgical wound infection outbreaks [13]. Recently, it was identified as the causative agent of respiratory outbreaks in two cystic fibrosis centers and showed evidence of patient-to-patient transmission [14, 15]. Infections with the *M. abscessus* group are difficult to treat, because these mycobacteria exhibit intrinsic resistance to various antibiotics. Susceptibility to clarithromycin (CLA) varies among members of the *M. abscessus* group; for example, while the majority of *M. abscessus* and *M. bolletii* exhibit resistance to CLA, *M. massiliense* is susceptible. Resistance is characterized by the presence of the *erm*(41) gene that encodes the erythromycin ribosomal methylases (ERM) [16].

Despite their close genetic relatedness, disparities in pathogenic potential, transmission mode, and antibiotic susceptibility were found among members of the *M. abscessus* group. Therefore, accurate identification of members of the *M. abscessus* group is important for patient treatment and epidemiological purposes [17].

Peptide nucleic acids (PNAs) are artificially synthesized DNA analogues with an uncharged peptide backbone. PNAs have more favorable hybridization properties and chemical, thermal, and biological stability due to their uncharged nature and peptide bond-linked backbone [18]. Due to these favorable characteristics, PNA has been widely applied as a diagnostic tool in molecular biology [19]. A PNA probe-based real-time PCR assay has been developed for mycobacteria diagnosis, particularly for the simultaneous separation of *M. tuberculosis* and NTM in clinical specimens [20, 21]. Recently, we introduced a novel peptide nucleic acid (PNA) multi-probe real- time PCR method that targeted the *hsp65* gene (*hsp65* PNA RT-PCR) to distinguish between the four types within the *M. abscessus* groups [*M. abscessus* and the 3 *M. massiliense* types (Type I, Type II-1 and Type II-2)] using 3 PNA probes. For this evaluation, we applied 27 reference strains and 228 clinical isolates belonging to the *M. abscessus* groups. With the exception of one clinical isolate, most of the samples (227/228 isolates, 99.6 % sensitivity) were clearly separated at the subspecies or genotype levels, thereby demonstrating the technique’s feasibility for the detection of *M. abscessus* at the mycobacterial culture level. To the best of our knowledge, this was the first report to use a PNA- based multi-probe approach for bacterial diagnosis [22].

The aim of this study was to evaluate the usefulness of the *hsp65* PNA RT-PCR in directly identifying the *M. abscessus* group at the subspecies or genotype levels from sputum samples. We applied the technique to sputum DNA samples from 60 different patients who were previously diagnosed with mycobacterial infections via *rpoB* PCR restriction analysis of the same cultures (30 samples were culture positive for *M. abscessus* complex strains, and 30 samples were culture positive for other mycobacteria). These results were compared with the results from the multi-probe real-time PCR assay targeting *hsp65* (HMPRT-PCR) that was previously developed by our laboratory [23].

## Methods

### Mycobacterial strains and sputum samples

Six mycobacteria reference strains including 5 strains belonging to the *M. abscessus* group (*M. abscessus* ATCC 19977^T^, *M. bolletii* CIP 108541^T^, *M. massiliense* Type I KCTC 19086^T^, *M. massiliense* Type II-1 SNUMC 50594 and *M. massiliense* Type II-2 SNUMC 53618) and *M. tuberculosis* H37Rv ATCC 27294^T^, and 60 sputum specimens suspected of harboring mycobacterial infection were used in this study. Four of the 6 mycobacterial reference strains were provided by the Korean Institute of Tuberculosis (KIT). *M. massiliense* Type II-1 SNUMC 50594 and *M. massiliense* Type II-2 SNUMC 53618 were provided by the Seoul National University College of Medicine (SNUMC). Sixty sputum samples from different patients with positive AFB smears detected between April 01, 2008, and July 31, 2008, at AMC, Seoul, Republic of Korea, were included in this study. Identification of mycobacterial infections in all sputum samples (30 samples were culture positive for *M. abscessus* complex strains and 30 samples were culture positive for other mycobacteria) was previously determined by *rpoB* PRA analysis of the same cultures. The sputa were digested, decontaminated, and concentrated as recommended by the WHO [24]. The processed sediment was stained using the Ziehl–Neelsen method. The results of the AFB smears were graded according to the recommendations of the American Thoracic Society and the Center for Disease Control and Prevention [25]. Sputa with trace AFB smear results (1–2 bacilli in 300 fields) were also included in this study. The protocol for this study including the documentation for waiver of informed consent was approved by the institutional review board of Seoul National University Hospital (C-1503-058-655) and Asan Medical Center (AMC IRB 2007–0331).

### DNA extraction

Chromosomal DNA was extracted from the sputum samples using the bead beater–phenol extraction method as previously described [26].

### *hsp65* PNA RT-PCR

For the detection of *M. abscessus* from sputum samples, the *hsp65* PNA RT-PCR method was applied to 60 sputum DNA samples, as previously described [22]. Briefly, a total of three reporter dyes were used for the specific simultaneous detection of the four types of the *M. abscessus* group (*M. abscessus*, Type I, Type II-1, and Type II-2) in a single reaction.: FAM for the detection of *M. abscessus* and *M. massiliense* at the species level, Hex for the discrimination of *M. massiliense* Type I and Type II, and Texas Red for the discrimination of *M. massiliense* Type II-1 and Type II-2. The sequences of the probes and primers are provided in Table 1. The probes were purchased from Panagene (Daejeon, Korea) and the primers from Macrogen (Seoul, Korea). A LightCycler (version 96; Roche Life Science, Mannheim, Germany) system was used for the real-time PCR, and three channels were used for the experiment. The optimal reaction mixture was established for the sensitive and specific detection of target sequences. A 10 μl reaction mixture was prepared for each sample as follows: 1 μl PCR reaction buffer for FastStart *Taq* (Roche), 2 mM MgCl_2_, 2 μl GC-RICH solution (Roche), 0.2 mM deoxynucleoside triphosphate mixture (Roche), 1 μM forward primer (Abs/Mas-F, 5′-CCGAGACGCTGCTGAAGAG-3′), 0.3 μM reverse primer (Abs/Mas-R, 5′-GACGTCCTCGGCGATGAT-3′), 0.4 μM PNA FAM probe (Mas/Abs, 5′-Dabcyl-CCTCGTTACCAACCT-O-K-FAM-3′), 0.15 μM PNA Hex probe (Mas-T2, 5′-Dabcyl-GGAGATTCCGGCC-O-K-Hex-3′), 0.5 μM PNA Texas Red probe (Mas-T2-1), 0.4 U FastStart Taq (Roche), 0.1 mg/ml bovine serum albumin (New England Biolabs), 2 μl template DNA, and distilled water (Roche). The cycling conditions were 300 s at 95 °C and 45 cycles of 20 s at 95 °C, 15 s at 62 °C (single acquisition of fluorescence signals), and 40 s at 74 °C. Melting curve analysis was performed using the following cycles: 120 s at 95 °C and 180 s at 37 °C with a ramping speed of 1.1 °C/s. Then, the temperature was increased from 37 °C to 80 °C at a temperature transition rate of 0.07 °C/s during which time the fluorescence signal was continuously acquired. Duplicate experiments were performed to determine the melting temperatures of the probes designed for the target *M. abscessus* group by real-time PCR, and DNA from a total of 60 sputum samples (Additional file 1) was subsequently tested for species identification by *T*_*m*_ analysis.

**Table 1:** Primers and PNA probes used for identification of *Mycobacterium abscessus* group in the present study

Primer/Probe	Sequence (5′ → 3′)	*T*_*m*_ (°C)^*a*^	Potential target organisms
*Primer*			
Abs/Mas-F	CCGAGACGCTGCTGAAGAG	66.2	*M. abscessus, M. massiliense*
Abs/Mas-R	GACGTCCTCGGCGATGAT	65.5	*M. abscessus, M. massiliense*
*PNA Probe*			
Mas/Abs	Dabcyl-CCTCGTTACCAACCT-O-K-FAM^*b*^	66.8	*M. massiliense, M. abscessus*
Mas-T2	Dabcyl-GGAGATTCCGGCC-O-K-Hex	69.5	*M. massiliense* Type 2/Type 1
Mas-T2-1	Dabcyl-CTGAATTACCTTCTC-O-K-Texas Red	62.0	*M. massiliense* Type 2-1/Type 2-2

^*a*^Primer *T*_*m*_ was calculated by using Oligo software V 6.50 and probe *T*_*m*_ was calculated by http://pnabio.com/support/PNA_Tool.htm; *T*_*m*_, melting temperature

^*b*^K, lysine; O, O linker

### HMPRT-PCR

The HMPRT-PCR was applied to the same 60 sputum DNA samples as previously described for comparison with the *hsp65* PNA RT-PCR [23]. Briefly, a total of 4 channels (CH.) were used for probes specific for 7 mycobacteria species: CH. 610 for the detection of *M. tuberculosis*, CH. 640 for the detection of *M. kansasii*, *M. intracellulare*, and *M. fortuitum-peregrinum* complex, CH. 670 for the detection of *M. avium*, and CH. 705 for the detection of *M. abscessus* and *M. massiliense*. A LightCycler 2.0 system was used for the real- time PCR. The LC Faststart DNA Master HP kit (Roche Diagnostics) was used for the preparation of the mastermix according to the kit protocol. A 10 μl reaction mixture was prepared for each sample as follows: 1 μl Taq buffer (containing dNTP mix and 10 mM MgCl_2_), an additional 2 mM MgCl_2_, 0.4 μM NTM-specific forward primer (HSP-NTM-F, 5′-CCGYTGCTGGAGAAGGTCATYCAG-3′), 0.3 μM TBC-specific forward primer (HSP-TBC-F, 5′-CGCTGCTCGAGAAGGTCATCGGA-3′), 1 μM mycobacteria-specific reverse primer (HSP-MYC-R, 5′-CGATGATGGTGGTCTCGTCCTTGGT-3′), 0.2 μM HybProbes, template (2 μl of culture-extracted DNA or 4 μl of sputum-extracted DNA with 0.1 mg/ml BSA (NEB)) and sterile distilled water. The cycling conditions were as follows: 10 min at 95 °C and 45 cycles of 10 s at 95 °C, 20 s at 65 °C (single acquisition of fluorescence signals) and 20 s at 72 °C. The cycling was followed by melting curve analysis: 10 s at 95 °C and 30 s at 57 °C. The temperature was increased from 57 °C to 90 °C with a temperature transition rate of 0.1 °C/s and the continuous acquisition of the fluorescence signal.

## Results

### Application of *hsp65* PNA RT-PCR to 30 sputum samples previously demonstrated to be infected by the *M. abscessus* group

**Fig. 1:**
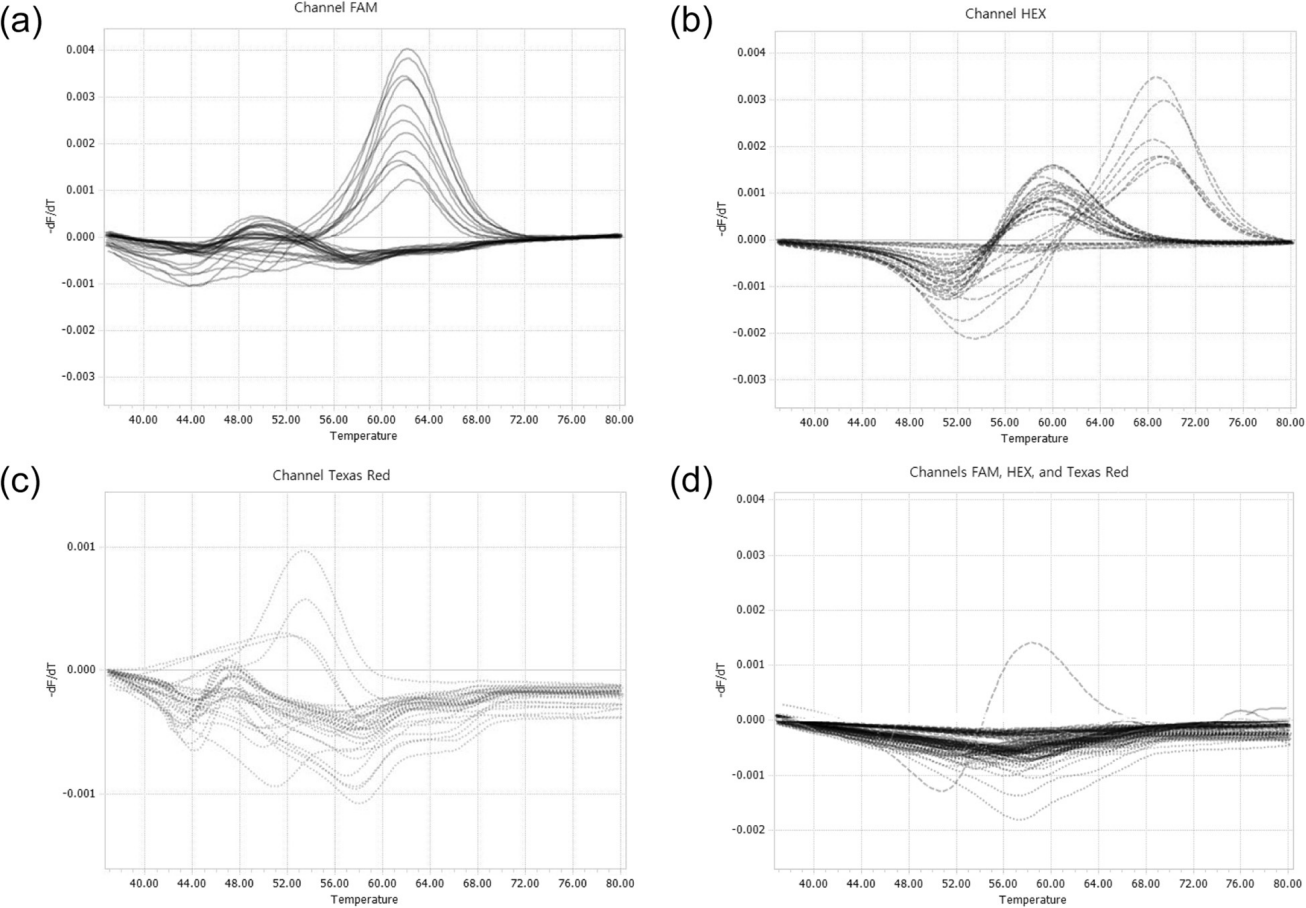
Melting curve analysis of the *hsp65* PNA real-time PCR of 60 sputa samples. *M. abscessus* and *M. massiliense* were differentially identified in the FAM channel (**a**), *M. massiliense* Type I and II in the HEX channel (**b**), and *M. massiliense* Type II-1 and II-2 in the Texas Red channel (**c**). Sputa including samples infected with non-*M. abscessus* group mycobacteria did not produce any significant melting peaks for the identification of the *M. abscessus* group genotypes in any of the three channels (**d**)

To evaluate the usefulness of *hsp65* PNA RT-PCR for the direct detection of *M. abscessus* group infection from sputa DNA, we tested a total of 30 sputum DNA samples from different patients previously diagnosed with infection by the *M. abscessus* group via *rpoB* PCR restriction analysis of the same cultures. The *hsp65* PNA RT-PCR method enabled the identification of 29 out of 30 sputum samples (96.7 %) into 18 *M. abscessus* and 11 *M. massiliense* strains. The assay was also able to differentiate all 11 *M. massiliense* strains into three genotypes: Type I (5 sputa), Type II-1 (4 sputa) and Type II-2 (2 sputa) (Fig. 1a-c, Table 2 and Additional file 1).

**Table 2:** Comparison of identification results between sputum *hsp65* PNA real-time PCR and sputum HMPRT-PCR based on same culture-based *rpoB* PRA

	*rpoB* PRA	HMPRT-PCR	*hsp65* PNA real-time PCR
*Sensitivity*			
*M. abscessus*	30	14	18
*M. massiliense*	NA	7	11
*M. massiliense* Type I	NA	NA	5
*M. massiliense* Type II-1	NA	NA	4
*M. massiliense* Type II-2	NA	NA	2
**Total No. of identification**		**21 (70 %)**	**29 (97 %)**
**No. of misidentification**		**0**	**0**
*Specificity*			
Non-*M. abscessus* group	30	28	0
*M. abscessus* group identified		**0 (100 %)**	**0 (100 %)**
**No. of misidentification**		**0**	**NA**

NA, not available

To verify the consistency of our *hsp65* PNA RT-PCR method in a clinical setting, we analyzed the range of *T*_*m*_s of the clinical samples at the intra-subspecies or intra-genotypic level in each channel. In the FAM channel, differences in *T*_*m*_s among the 18 *M. abscessus* sputum samples and 11 *M. massiliense* strains were 2.9 °C (49.0–52.3 °C) and 0.7 °C (61.4–62.1 °C), respectively, which was less than the inter-subspecies *T*_*m*_s differences between *M. abscessus* and *M. massiliense* (11.5–11.9 °C). In the Hex channel, differences in *T*_*m*_s among the 4 *M. massiliense* Type I and 6 *M. massiliense* Type II sputum samples were 1.0 °C (60.0–61.0 °C) and 1.1 °C (68.5–69.6 °C), respectively, which was less than the inter-subspecies *T*_*m*_s difference between *M. massiliense* Type I and Type II (8.8–9.1 °C). In the Texas Red channel, the differences in *T*_*m*_s among the 4 *M. massiliense* Type II-1 and 2 *M. massiliense* Type II-2 sputum samples were 1.9 °C (51.5–53.4 °C) and 0.3 °C (46.4–46.7 °C), respectively, which was less than the inter-genotypic *T*_*m*_s difference between *M. massiliense* Type II-1 and Type II-2 (4.8–7 °C) (Table 3). These results demonstrate the suitability of the *hsp65* PNA RT-PCR for the separation of the *M. abscessus* group into subspecies or genotypes in clinical settings.

**Table 3:** Measurement of melting temperatures of *M. abscessus* group from sputa samples by *hsp65* PNA real-time PCR

	Channels		
Species	FAM	Hex	Texas Red
*M. abscessus*			
No. of identification	18	18	18
Average *T*_*m*_ (°C)^*b*^	**50.0 ± 0.85**	59.7 ± 0.37	47.1 ± 0.45
Range of *T*_*m*_ (°C)	**49.0**–**52.3**	59.0–60.5	46.3–47.7
No. of failed detection	0	0	3
*M. massiliense* Type I			
No. of identification	5	5	5
Average *T*_*m*_ (°C)	**61.8 ± 0.37**	60.6 ± 0.56	46.9 ± 0.28
Range of *T*_*m*_ (°C)	**61.4**–**62.1**	59.9–61.0	46.5–47.2
No. of failed detection	0	0	0
*M. massiliense* Type II-1			
No. of identification	4	4	4
Average *T*_*m*_ (°C)	**61.9 ± 0.08**	**69.1 ± 0.49**	**52.7 ± 0.89**
Range of *T*_*m*_ (°C)	**62.0**–**63.3**	**68.4**–**71.3**	**51.5**–**53.4**
No. of failed detection	0	0	0
*M. massiliense* Type II-2			
No. of identification	2	2	2
Average *T*_*m*_ (°C)	**62.0 ± 0.07**	**69.0 ± 0.42**	**46.6 ± 0.21**
Range of *T*_*m*_ (°C)	**61.9**–**62.0**	**68.7**–**69.3**	**46.4**–**46.7**
No. of failed detection	0	0	0

^*a*^mean ± SD

^*b*^ *T*_*m*_, melting temperature; boldface, species specific *T*_*m*_; −, no significant *T*_*m*_

### Application of *hsp65* PNA RT-PCR to 30 sputum samples previously demonstrated to be infected by mycobacteria other than the *M. abscessus* group

To validate the specificity of the *hsp65* PNA RT-PCR method in detecting *M. abscessus* group infections from sputum samples, we applied the technique to 30 sputum samples from different patients previously identified as infected with mycobacteria other than the *M. abscessus* group (10 patients with *M. tuberculosis*, 9 with *M. avium*, 7 with *M. intracellulare*, 1 with *M. celatum*, 1 with *M. fortuitum*, 1 with *M. kansasii* and 1 with *M. szulgai*) via *rpoB* PCR restriction analysis of the same cultures. None of the 30 sputum DNA samples produced a detectable *T*_*m*_ in the FAM channel that was specific for *M. abscessus* group species detection, thereby validating the high specificity of the *hsp65* PNA RT-PCR method for the detection of the *M. abscessus* group from sputum samples. Only one sputum sample (*M. fortuitum*) formed an insignificant *T*_*m*_ in the Hex channel (Fig. 1d).

### Comparison of *hsp65* PNA RT-PCR versus HMPRT-PCR for the direct detection of the *M. abscessus* group from sputum samples

Previously, we introduced a multiprobe real-time PCR assay targeting *hsp65* (HMPRT-PCR) to detect and identify pathogenic mycobacteria directly from sputum specimens. In this study, we compared the 2 methods (*hsp65* PNA RT-PCR and HMPRT-PCR) for the direct detection of the *M. abscessus* group from sputum samples. When the 2 methods were applied to the same 30 sputum DNA samples, the results showed that the *hsp65* PNA RT-PCR had higher sensitivity compared to the HMPRT-PCR in detecting *M. abscessus* from sputum [96.7 % (29/30 samples) vs. 70 % (21/30samples)] (Table 2). A discrepancy between the two methods was found in nine samples. One sputum sample (S1380) identified as *M. massiliense* Type II-1 infection by *hsp65* PNA RT-PCR was identified as *M. avium* infection by HMPRT-PCR; its culture identification was confirmed to be a coinfection of *M. abscessus* and *M. avium*. HMPRT-PCR failed to detect *M. abscessus* in this coinfection. Another sample (S1479) identified as *M. massiliense* Type II-2 infection by *hsp65* PNA RT-PCR was identified as a coinfection of *M. massiliense* and *M. avium* HMPRT-PCR; its culture identification was *M. abscessus* infection. HMPRT-PCR could not detect 5 samples identified as *M. abscessus* infections by *hsp65* PNA RT-PCR or two samples identified as *M. massiliense* Type I and Type II-1 infections by *hsp65* PNA RT-PCR (Additional file 1).

## Discussion

Recently, we reported that phylogenetic analysis based on the sequence of the 604-bp *hsp65* gene enabled taxonomic separation among members of the *M. abscessus* groups at the genotype and subspecies levels despite representing a single target gene [9]. We also reported that the separation of members of the *M. abscessus* group based on pulsed-field gel electrophoresis (PFGE) and multi-locus sequence typing (MLST) was nearly in agreement with the results obtained using sequence-based phylogenetic analysis of the 604-bp *hsp65* gene [12], which resulted in the phylogenetic separation of four members of the *M. abscessus* groups [*M. abscessus* and the three types of *M. massiliense* (I, II-1 and II-2)].

SNPs have been widely used for the diagnostic targeting of pathogens [27, 28] and human diseases [29]. For use in bacterial identification, an SNP should show not only intra-species or genotypic conservation but also inter-species or genotypic variation. Notably, there are several SNPs in the 604-bp *hsp65* sequence that are potential targets of diagnostic methods for the separation of members of *M. abscessus*. In a previous study, we developed an *hsp65* PNA RT-PCR targeting three signature SNPs in the 604-bp *hsp65* gene that enabled the separation of the four types of *M. abscessus* (*M. abscessus* and three types of *M. massiliense*) at the cultured isolate level. The intra-genotypic conservation and inter-genotypic variation for each SNP of members of the *M. abscessus* group were confirmed by successful separation in 227 out of 228 clinical isolates (99.6 %) [22]. In this study, the application of the *hsp65* PNA RT-PCR method to sputum samples demonstrated that this method could directly identify members of the *M. abscessus* group at the subspecies or genotypic level from sputum samples with 96.7 % sensitivity (29/30 samples) and 100 % specificity (Table 2), thereby demonstrating its feasibility in identifying the *M. abscessus* group not only from cultured isolates but also directly from sputum samples.

PNA-based molecular beacons were reported to be superior to conventional molecular probes due to their faster hybridization kinetics, high signal to background ratio and improved specificity [30]. Indeed, PNAs have been successfully applied for the highly sensitive detection of anthrax DNA and HIV RNA [31, 32]. PNA-based molecular beacons were reported to be advantageous for genotyping short sequences when high sequence specificity was required. The typing of an SNP with DNA probes such as the TaqMan or LightCycler probes (which are usually at least 23 nucleotides in length) can be problematic due to the limited discriminating power of long DNA probes. PNA molecular probes are significantly shorter than the TaqMan or LightCycler probes, making probe design and genotype discrimination easier [33]. Despite the short sequence length (13–15 bp) of the probes, our *hsp65* PNA RT-PCR could be successfully applied to the separation of members of the *M. abscessus* group from sputum samples (Table 1) as well as clinical isolates [22]. The presence of a mismatch in a PNA/DNA duplex is reported to be more destabilizing than a mismatch in a DNA/DNA duplex, suggesting that the use of PNA probes may lead to more pronounced difference in the average *T*_*m*_ in SNP detection compared to the use of DNA probes [34]. In this study, our PNA probes showed relatively large differences (4.8 °C to 11.9 °C) in *T*_*m*_s in SNP detection despite one nucleotide mismatch. Thus, our probes provided improved discriminative power for the separation of members of the *M. abscessus* group compared to DNA probes, which are always higher compared to the probes used for intra-subspecies or intra-genotype analyses. This result demonstrates the feasibility and reproducibility of our *hsp65* PNA RT-PCR method in a clinical setting. The comparison of *hsp65* PNA RT-PCR versus HMPRT-PCR in this study demonstrated the higher sensitivity of the former compared to the latter [96.7 % (29/30 samples) vs. 70 % (21/30 samples)] in detecting *M. abscessus* from sputum samples. These findings strongly support the hypothesis that PNA-probe based technology may be superior to technologies based on other probes (i.e., FRET-based dual probes for the direct detection of mycobacteria from sputum samples).

Notably, the *T*_*m*_s measured from sputum samples showed small decreasing shifts (1–2.7 °C) compared to our previously reported *T*_*m*_s of clinical isolates of the *M. abscessus* genotypes [22] without compromising the discrimination of the genotypes. This outcome may be due to the presence of PCR-inhibitory substances in the DNA extracts from the sputum specimens because inhibitory substances can cause *T*_*m*_ shifts [35, 36].

## Conclusions

In conclusion, our data suggest that the *hsp65* PNA RT-PCR method developed in the previous study may represent a promising approach for identifying the subspecies or genotypes of the *M. abscessus* group directly from sputum samples.

## Additional file

Additional file 1:
**Identification of** ***M. abscessus*** **and** ***M. massiliense*** **genotypes from DNA extracts of sputa samples using** ***hsp65*** **PNA real-time PCR.** (DOCX 19 kb)

## Abbreviations

AFB: Acid-fast bacillus
CLA: Clarithromycin
ERM: Erythromycin ribosomal methylase
GPL: Glycopeptidolipid
KIT: Korean Institute of Tuberculosis
MLST: Multi-locus sequence typing
NTM: Nontuberculous mycobacteria
PFGE: Pulsed-field gel electrophoresis
PNA: Peptide nucleic acid
PRA: PCR-restriction enzyme analysis
RGM: Rapidly growing mycobacteria
SNP: Single nucleotide polymorphism
SNUMC: Seoul National University College of Medicine
