# Supplementary material for: Separation of Mycobacterium abscessus into subspecies or genotype level by direct application of peptide nucleic acid multi-probe- real-time PCR method into sputa samples
Source: BMC Infect Dis. 2015 Aug 11;15:325. doi: 10.1186/s12879-015-1076-8 (PMC4531893; doi:10.1186/s12879-015-1076-8)
Supplement: Additional file 1: — Identification of M. abscessus and M. massiliense genotypes from DNA extracts of sputa samples using hsp65 PNA real-time PCR. (DOCX 19 kb) [file 12879_2015_1076_MOESM1_ESM.docx]

**Additional file 1.** Identification of *M. abscessus* and *M. massiliense* genotypes from DNA extracts of sputa samples by *hsp65* PNA real-time PCR

|  |  |  |  | *hsp65* PNA real-time PCR | | | | |
| --- | --- | --- | --- | --- | --- | --- | --- | --- |
|  |  |  |  |  | Measured *T_m_* (°C) | | |  |
| No. | Sputum ID | *rpoB* PRA identification | HMPRT-PCR identification | Copy No. | FAM | Hex | Texas Red | Identification |
| 1 | 814 | *M. abscessus* | *M. abscessus* | 40.4 | **50.2** | 60.0 | 47.7 | *M. abscessus* |
| 2 | 876 | *M. abscessus* | *M. massiliense* | 126.5 | **62.1** | 61.0 | 46.8 | *M. massiliense* Type I |
| 3 | 984 | *M. abscessus* | *M. abscessus* | 4.0 | **49.9** | 60.2 | 47.5 | *M. abscessus* |
| 4 | 993 | *M. abscessus* | *M. abscessus* | 17.4 | **50.1** | 60.5 | 47.1 | *M. abscessus* |
| 5 | 1021 | *M. abscessus* or *M. intracellulare* | *M. abscessus* | 4.6 | **49.9** | 60.1 | 47.5 | *M. abscessus* |
| 6 | 1039 | *M. abscessus* | - | 17.6 | **49.8** | 59.8 | 46.9 | *M. abscessus* |
| 7 | 1085 | *M. abscessus* | *M. abscessus* | 8.7 | **49.9** | 59.6 | 47.5 | *M. abscessus* |
| 8 | 1089 | *M. abscessus* | *M. massiliense* | 270.2 | **61.9** | **69.6** | **53.3** | *M. massiliense* Type II-1 |
| 9 | 1093 | *M. abscessus* | *M. abscessus* | 13.2 | **50.0** | 60.1 | 47.4 | *M. abscessus* |
| 10 | 1112 | *M. abscessus* | *M. abscessus* | 0.3 | **49.8** | 59.7 | - | *M. abscessus* |
| 11 | 1124 | *M. abscessus* | *M. massiliense* | 93.8 | **62.0** | **68.7** | **46.4** | *M. massiliense* Type II-2 |
| 12 | 1126 | *M. abscessus* | - | 5.7 | **49.7** | 59.9 | 46.8 | *M. abscessus* |
| 13 | 1257 | *M. abscessus* | - | 118.6 | **62.0** | **69.3** | **52.4** | *M. massiliense* Type II-1 |
| 14 | 1276 | *M. abscessus* | *M. abscessus* | 0.2 | **49.7** | 59.8 | - | *M. abscessus* |
| 15 | 1279 | *M. abscessus* | - |  | **-** | - | - | - |
| 16 | 1287 | *M. abscessus* | *M. abscessus* | 0.4 | **49.4** | 59.5 | 46.5 | *M. abscessus* |
| 17 | 1295 | *M. abscessus* | *M. abscessus* | 2.6 | **52.3** | 59.2 | 46.6 | *M. abscessus* |
| 18 | 1315 | *M. abscessus* | - | 0.2 | **49.4** | 59.5 | 47.4 | *M. abscessus* |
| 19 | 1326 | *M. abscessus* | *M. abscessus* | 87.3 | **49.4** | 59.9 | 46.3 | *M. abscessus* |
| 20 | 1347 | *M. abscessus* | *M. massiliense* | 77.3 | **62.0** | 60.9 | 47.2 | *M. massiliense* Type I |
| 21 | 1349 | *M. abscessus* | - | 0.4 | **61.4** | 59.9 | 46.8 | *M. massiliense* Type I |
| 22 | 1359 | *M. abscessus* | - | 3.4 | **49.4** | 59.4 | 47.3 | *M. abscessus* |
| 23 | 1371 | *M. abscessus* | *M. abscessus* | 7.5 | **49.4** | 59.6 | 47.4 | *M. abscessus* |
| 24 | 1380 | *M. abscessus, M. avium* | *M. avium* | 48.9 | **61.8** | **68.8** | **53.4** | *M. massiliense* Type II-1 |
| 25 | 1428 | *M. abscessus* | *M. abscessus* | 0.1 | **52.0** | 59.0 | - | *M. abscessus* |
| 26 | 1479 | *M. abscessus* | *M. massiliense, M. avium* | 548.3 | **61.9** | **69.3** | **46.7** | *M. massiliense* Type II-2 |
| 27 | 1480 | *M. abscessus* | - | 0.1 | **49.0** | 59.5 | 46.5 | *M. abscessus* |
| 28 | 1491 | *M. abscessus* | *M. massiliense* | 315.7 | **62.1** | 61.0 | 47.1 | *M. massiliense* Type I |
| 29 | 1495 | *M. abscessus* | *M. massiliense* | 9.4 | **61.9** | **68.5** | **51.5** | *M. massiliense* Type II-1 |
| 30 | 1497 | *M. abscessus* | *M. abscessus* | 0.7 | **61.4** | 60.0 | 46.5 | *M. massiliense* Type I |
| 31 | 775 | *M. intracellulare* | *M. intracellulare* |  | - | - | - | - |
| 32 | 791 | *M. intracellulare* | *M. intracellulare* |  | - | - | - | - |
| 33 | 811 | *M. avium* | *M. avium* |  | - | - | - | - |
| 34 | 902 | *M. avium* | *M. avium* |  | - | - | - | - |
| 35 | 1044 | *M. avium* | *M. avium* |  | - | - | - | - |
| 36 | 1073 | *M. tuberculosis* | *M. tuberculosis* |  | - | - | - | - |
| 37 | 1074 | *M. tuberculosis* | *M. tuberculosis* |  | - | - | - | - |
| 38 | 1075 | *M. tuberculosis* | *M. tuberculosis* |  | - | - | - | - |
| 39 | 1079 | *M. tuberculosis* | *M. tuberculosis* |  | - | - | - | - |
| 40 | 1080 | *M. tuberculosis* | *M. tuberculosis* |  | - | - | - | - |
| 41 | 1086 | *M. intracellulare* | *M. intracellulare* |  | - | - | - | - |
| 42 | 1088 | *M. tuberculosis* | *M. tuberculosis* |  | - | - | - | - |
| 43 | 1099 | *M. tuberculosis* | *M. tuberculosis* |  | - | - | - | - |
| 44 | 1100 | *M. intracellulare* | *M. intracellulare* |  | - | - | - | - |
| 45 | 1119 | *M. intracellulare* | *M. intracellulare* |  | - | - | - | - |
| 46 | 1267 | *M. avium* | *M. avium* |  | - | - | - | - |
| 47 | 1298 | *M. intracellulare* | *M. intracellulare* |  | - | - | - | - |
| 48 | 1304 | *M. fortuitum* | *M. fortuitum* |  | - | 58.4 | - | - |
| 49 | 1306 | *M. intracellulare* | *M. intracellulare* |  | - | - | - | - |
| 50 | 1322 | *M. celatum* | - |  | - | - | - | - |
| 51 | 1381 | *M. avium* | *M. avium* |  | - | - | - | - |
| 52 | 1390 | *M. avium* | *M. avium* |  | - | - | - | - |
| 53 | 1414 | *M. avium* | *M. avium* |  | - | - | - | - |
| 54 | 1427 | *M. avium* | *M. avium* |  | - | - | - | - |
| 55 | 1432 | *M. avium* | *M. avium* |  | - | - | - | - |
| 56 | 1450 | *M. kansasii* | *M. kansasii* |  | - | - | - | - |
| 57 | 1483 | *M. tuberculosis* | *M. tuberculosis* |  | - | - | - | - |
| 58 | 1484 | *M. szulgai* | - |  | - | - | - | - |
| 59 | 1485 | *M. tuberculosis* | *M. tuberculosis* |  | - | - | - | - |
| 60 | 1486 | *M. tuberculosis* | *M. tuberculosis* |  | - | - | - | - |

*T_m_*, melting temperature; boldface, genotype-specific *T_m_*; -, not detected or not identified
